# Supplementary material for: A fault-intrusion-tolerant system and deadline-aware algorithm for scheduling scientific workflow in the cloud
Source: PeerJ Comput Sci. 2021 Nov 2;7:e747. doi: 10.7717/peerj-cs.747 (PMC8576554; doi:10.7717/peerj-cs.747)
Supplement: Supplemental Information 6 [file peerj-cs-07-747-s006.zip › Myproject1.1/docs/index-files/index-6.html]

F-Index


JavaScript is disabled on your browser.


- Overview
- Package
- Class
- Use
- Tree
- Deprecated
- Index
- Help

- Prev Letter
- Next Letter

- Frames
- No Frames

- All Classes

A B C D E F G H I J L M N O P Q R S T U V W Z 


## F

FAILED - Static variable in class org.cloudbus.cloudsim.Cloudlet
:   The Cloudlet is failed.

FAILED - Static variable in class org.cloudbus.cloudsim.Pe
:   Denotes Pe is failed and hence it can't process any Cloudlet at this moment.

FAILED\_RESOURCE\_UNAVAILABLE - Static variable in class org.cloudbus.cloudsim.Cloudlet
:   The cloudlet has failed due to a resource failure.

failedTasksNum - Variable in class org.workflowsim.failure.FailureRecord
:   number of failed tasks.

FailureGenerator - Class in org.workflowsim.failure
:   FailureGenerator creates a failure when a job returns

FailureGenerator() - Constructor for class org.workflowsim.failure.FailureGenerator


FailureMonitor - Class in org.workflowsim.failure
:   FailureMonitor collects failure information

FailureMonitor() - Constructor for class org.workflowsim.failure.FailureMonitor


FailureRecord - Class in org.workflowsim.failure
:   Failure Record is a record with all information of a failure

FailureRecord(double, int, int, int, int, int, int) - Constructor for class org.workflowsim.failure.FailureRecord
:   Initialize a Failure Record

FALSE - Static variable in class org.cloudbus.cloudsim.core.CloudSimTags
:   Denotes boolean false in int value

FCFSScheduler - Class in org.workflowsim.scheduler
:   The FCFS algorithm.

FCFSScheduler() - Constructor for class org.workflowsim.scheduler.FCFSScheduler


File - Class in org.cloudbus.cloudsim
:   A class for representing a physical file in a DataCloud environment

File(String, int) - Constructor for class org.cloudbus.cloudsim.File
:   Creates a new DataCloud file with a given size (in MBytes).

File(File) - Constructor for class org.cloudbus.cloudsim.File
:   Copy constructor, i.e.

FILE\_ADD\_ERROR - Static variable in class org.cloudbus.cloudsim.DataCloudTags
:   Denotes that file addition is failed due to an unknown error

FILE\_ADD\_ERROR\_ACCESS\_DENIED - Static variable in class org.cloudbus.cloudsim.DataCloudTags
:   Denotes that file addition is failed because access/permission denied or not authorized

FILE\_ADD\_ERROR\_EMPTY - Static variable in class org.cloudbus.cloudsim.DataCloudTags
:   Denotes that file addition is failed because the given file is empty

FILE\_ADD\_ERROR\_EXIST\_READ\_ONLY - Static variable in class org.cloudbus.cloudsim.DataCloudTags
:   Denotes that file addition is failed because the file already exists in the catalogue and it
    is read-only file

FILE\_ADD\_ERROR\_STORAGE\_FULL - Static variable in class org.cloudbus.cloudsim.DataCloudTags
:   Denotes that file addition is failed because the storage is full

FILE\_ADD\_MASTER - Static variable in class org.cloudbus.cloudsim.DataCloudTags
:   Requests for a master file to be added to the local storage(s).  
    The format of this request is Object[2] = {File obj, Integer senderID}.  
    The reply tag name is `DataCloudTags.FILE_ADD_MASTER_RESULT`.

FILE\_ADD\_MASTER\_RESULT - Static variable in class org.cloudbus.cloudsim.DataCloudTags
:   Sends the result of adding a master file back to sender.  
    The format of the reply is Object[3] = {String lfn, Integer uniqueID, Integer resultID}.  
    NOTE: The result id is in the form of FILE\_ADD\_XXXX where XXXX means the error/success
    message

FILE\_ADD\_REPLICA - Static variable in class org.cloudbus.cloudsim.DataCloudTags
:   Requests for a replica file to be added from the local storage(s).  
    The format of this request is Object[2] = {File obj, Integer senderID}.  
    The reply tag name is `DataCloudTags.FILE_ADD_REPLICA_RESULT`.

FILE\_ADD\_REPLICA\_RESULT - Static variable in class org.cloudbus.cloudsim.DataCloudTags
:   Sends the result of adding a replica file back to sender.  
    The format of the reply is Object[2] = {String lfn, Integer resultID}.  
    NOTE: The result id is in the form of FILE\_ADD\_XXXX where XXXX means the error/success
    message

FILE\_ADD\_SUCCESSFUL - Static variable in class org.cloudbus.cloudsim.DataCloudTags
:   Denotes that file addition is successful

FILE\_DELETE\_ERROR - Static variable in class org.cloudbus.cloudsim.DataCloudTags
:   Denotes that file deletion is failed due to an unknown error

FILE\_DELETE\_ERROR\_ACCESS\_DENIED - Static variable in class org.cloudbus.cloudsim.DataCloudTags
:   Denotes that file deletion is failed because access/permission denied or not authorized

FILE\_DELETE\_ERROR\_DOESNT\_EXIST - Static variable in class org.cloudbus.cloudsim.DataCloudTags
:   Denotes that file deletion is failed because the file does not exist in the storage nor
    catalogue

FILE\_DELETE\_ERROR\_IN\_USE - Static variable in class org.cloudbus.cloudsim.DataCloudTags
:   Denotes that file deletion is failed because it is currently used by others

FILE\_DELETE\_ERROR\_READ\_ONLY - Static variable in class org.cloudbus.cloudsim.DataCloudTags
:   Denotes that file deletion is failed because it is a read-only file

FILE\_DELETE\_MASTER - Static variable in class org.cloudbus.cloudsim.DataCloudTags
:   Requests for a master file to be deleted from the local storage(s).  
    The format of this request is Object[2] = {String lfn, Integer senderID}.  
    The reply tag name is `DataCloudTags.FILE_DELETE_MASTER_RESULT`.

FILE\_DELETE\_MASTER\_RESULT - Static variable in class org.cloudbus.cloudsim.DataCloudTags
:   Sends the result of deleting a master file back to sender.  
    The format of the reply is Object[2] = {String lfn, Integer resultID}.  
    NOTE: The result id is in the form of FILE\_DELETE\_XXXX where XXXX means the error/success
    message

FILE\_DELETE\_REPLICA - Static variable in class org.cloudbus.cloudsim.DataCloudTags
:   Requests for a replica file to be deleted from the local storage(s).  
    The format of this request is Object[2] = {String lfn, Integer senderID}.  
    The reply tag name is `DataCloudTags.FILE_DELETE_REPLICA_RESULT`.

FILE\_DELETE\_REPLICA\_RESULT - Static variable in class org.cloudbus.cloudsim.DataCloudTags
:   Sends the result of deleting a replica file back to sender.  
    The format of the reply is Object[2] = {String lfn, Integer resultID}.  
    NOTE: The result id is in the form of FILE\_DELETE\_XXXX where XXXX means the error/success
    message

FILE\_DELETE\_SUCCESSFUL - Static variable in class org.cloudbus.cloudsim.DataCloudTags
:   Denotes that file deletion is successful

FILE\_DELIVERY - Static variable in class org.cloudbus.cloudsim.DataCloudTags
:   Sends the file to the requester.

FILE\_MODIFY - Static variable in class org.cloudbus.cloudsim.DataCloudTags
:   Requests for a file to be modified from the local storage(s).  
    The format of this request is Object[2] = {File obj, Integer senderID}.  
    The reply tag name is `DataCloudTags.FILE_MODIFY_RESULT`.

FILE\_MODIFY\_ERROR - Static variable in class org.cloudbus.cloudsim.DataCloudTags
:   Denotes that file modification is failed due to an unknown error

FILE\_MODIFY\_ERROR\_ACCESS\_DENIED - Static variable in class org.cloudbus.cloudsim.DataCloudTags
:   Denotes that file modification is failed because access/permission denied or not authorized

FILE\_MODIFY\_ERROR\_DOESNT\_EXIST - Static variable in class org.cloudbus.cloudsim.DataCloudTags
:   Denotes that file modification is failed because the file does not exist

FILE\_MODIFY\_ERROR\_IN\_USE - Static variable in class org.cloudbus.cloudsim.DataCloudTags
:   Denotes that file modification is failed because the file is currently used by others

FILE\_MODIFY\_ERROR\_READ\_ONLY - Static variable in class org.cloudbus.cloudsim.DataCloudTags
:   Denotes that file modification is failed because it is a read-only file

FILE\_MODIFY\_RESULT - Static variable in class org.cloudbus.cloudsim.DataCloudTags
:   Sends the result of deleting a file back to sender.  
    The format of the reply is Object[2] = {String lfn, Integer resultID}.  
    NOTE: The result id is in the form of FILE\_MODIFY\_XXXX where XXXX means the error/success
    message

FILE\_MODIFY\_SUCCESSFUL - Static variable in class org.cloudbus.cloudsim.DataCloudTags
:   Denotes that file modification is successful

FILE\_REQUEST - Static variable in class org.cloudbus.cloudsim.DataCloudTags
:   Requests for a file that is stored on the local storage(s).  
    The format of this request is Object[2] = {String lfn, Integer senderID}.  
    The reply tag name is `DataCloudTags.FILE_DELIVERY`.

FILE\_SIZE - Static variable in class org.cloudbus.cloudsim.network.datacenter.NetworkConstants


FileAttribute - Class in org.cloudbus.cloudsim
:   A class for storing related information regarding to a `gridsim.datagrid.File` entity.

FileAttribute(String, int) - Constructor for class org.cloudbus.cloudsim.FileAttribute
:   Allocates a new FileAttribute class.

finalizeCloudlet() - Method in class org.cloudbus.cloudsim.ResCloudlet
:   Finalizes all relevant information before exiting the CloudResource entity.

findFirstDeferred(int, Predicate) - Static method in class org.cloudbus.cloudsim.core.CloudSim
:   Find first deferred event matching a predicate.

findHostForVm(Vm) - Method in class org.cloudbus.cloudsim.power.PowerVmAllocationPolicyAbstract
:   Find host for vm.

findHostForVm(Vm, Set<? extends Host>) - Method in class org.cloudbus.cloudsim.power.PowerVmAllocationPolicyMigrationAbstract
:   Find host for vm.

findHostForVm(Vm) - Method in class org.cloudbus.cloudsim.power.PowerVmAllocationPolicyMigrationAbstract
:   Find host for vm.

FINISH - Static variable in class org.cloudbus.cloudsim.network.datacenter.NetworkConstants


FINISHED - Static variable in class org.cloudbus.cloudsim.core.SimEntity
:   The Constant FINISHED.

finishExecution() - Method in class org.cloudbus.cloudsim.DatacenterBroker
:   Send an internal event communicating the end of the simulation.

finishExecution() - Method in class org.workflowsim.ClusteringEngine
:   Send an internal event communicating the end of the simulation.

finishExecution() - Method in class org.workflowsim.WorkflowPlanner
:   Send an internal event communicating the end of the simulation.

finishSimulation() - Static method in class org.cloudbus.cloudsim.core.CloudSim
:   Internal method that allows the entities to terminate.

finishtime - Variable in class org.cloudbus.cloudsim.network.datacenter.NetworkCloudlet


finishtime - Variable in class org.cloudbus.cloudsim.network.datacenter.NetworkVm


fintime - Variable in class org.cloudbus.cloudsim.network.datacenter.NetworkHost


fintimelistHost - Variable in class org.cloudbus.cloudsim.network.datacenter.Switch


fintimelistVM - Variable in class org.cloudbus.cloudsim.network.datacenter.Switch


flagfree - Variable in class org.cloudbus.cloudsim.network.datacenter.NetworkVm


FloydWarshall\_Float - Class in org.cloudbus.cloudsim.network
:   FloydWarshall algorithm to calculate all pairs delay and predecessor matrix.

FloydWarshall\_Float() - Constructor for class org.cloudbus.cloudsim.network.FloydWarshall\_Float


format(String, Object...) - Static method in class org.cloudbus.cloudsim.Log
:   Prints a string formated as in String.format().

formatLine(String, Object...) - Static method in class org.cloudbus.cloudsim.Log
:   Prints a line formated as in String.format().

FREE - Static variable in class org.cloudbus.cloudsim.Pe
:   Denotes Pe is FREE for allocation.

future - Static variable in class org.cloudbus.cloudsim.core.CloudSim
:   The future event queue.

FutureQueue - Class in org.cloudbus.cloudsim.core
:   This class implements the future event queue used by `Simulation`.

FutureQueue() - Constructor for class org.cloudbus.cloudsim.core.FutureQueue

A B C D E F G H I J L M N O P Q R S T U V W Z

- Overview
- Package
- Class
- Use
- Tree
- Deprecated
- Index
- Help

- Prev Letter
- Next Letter

- Frames
- No Frames

- All Classes
